# Application of Exercise/Training Models to Evaluate Food Functionality with Special Focus on Preventing Inflammation and Oxidative Stress and Enhancing Exercise Performance

**DOI:** 10.3390/foods14234025

**Published:** 2025-11-24

**Authors:** Katsuhiko Suzuki, Cong Wu, Sihui Ma

**Affiliations:** 1 Faculty of Sport Sciences, Waseda University, Tokorozawa 359-1192, Saitama, Japan; 2 Graduate School of Sport Sciences, Waseda University, Tokorozawa 359-1192, Saitama, Japan

**Keywords:** exercise models, functional foods, inflammation, oxidative stress, athletic performance, sports nutrition

## Abstract

Exercise and physical training induce diverse physiological responses that can be modulated by functional foods. This article examines how different exercise models—from moderate exercise to intense training—can be applied to evaluate food functionality in preventing inflammation, oxidative stress, and enhancing athletic performance. We discuss the paradoxical nature of exercise, where moderate physical activity promotes health through anti-inflammatory and antioxidant effects, while intense exercise can induce muscle damage, inflammation, and immunosuppression. Through analysis of recent research, including studies on polyphenols, amino acid derivatives, and novel delivery systems, we highlight the importance of appropriate exercise model selection, timing, and dosage of nutritional interventions. Emerging approaches such as nano-processed compounds, gut microbiota modulation, and synergistic combinations offer promising strategies. This review provides guidance for researchers and practitioners in selecting suitable exercise models to evaluate functional foods, emphasizing the need for personalized approaches that balance performance enhancement with health protection.

## 1. Introduction

Moderate exercise is useful for improving health and preventing disease, but intense exercise and training can cause muscle pain, fatigue, and other health problems that can deteriorate conditions and lead to poor performance [1,2,3]. In addition, the range of exercise is wide, from moderate exercise at the level of exercise prescription to intense exercise such as running a marathon, as well as various types of training such as those performed by athletes. The use of foods in each situation is also wide-ranging. The type, dosage, and timing of food intake need to be considered according to the characteristics of the athletic events, such as sprint, endurance, and power, the season of competition, and the conditions before and after training and/or competition. To combine these various conditions and evaluate biological effects, various exercise models have been used in research, including walking or cycling [4,5,6] as a moderate exercise and voluntary running exercise [7,8,9] in animal experiments. Intense exercise, such as the maximal exercise stress test [10,11,12,13], long-distance running [14,15,16,17], and, in animal experiments, exhaustive exercise [18,19,20,21], is frequently used. Since there are various types of exercise and training, it is necessary to use different foods for different purposes, and to be careful to consume the appropriate amount of food that expresses functionality and does not constitute doping. In this paper, we introduce specific exercise models and biomarkers that are useful for evaluation of prevention, early recovery, and performance evaluation of muscle injury, visceral injury, inflammation, oxidative stress, fatigue, and immunodepression caused by intense exercise and training [1,22], along with light exercise effectiveness on anti-inflammatory and antioxidant actions [2].

## 2. Moderate Exercise

Exercise for health promotion typically involves sessions of 20–60 min at moderate intensity, where safety considerations often take precedence over performance outcomes and the workloads are relatively low [2,22]. This exercise domain provides an ideal platform for evaluating the long-term health benefits of functional foods, as inflammation and oxidative stress responses are generally mild and manageable.

Recent investigations have revealed interesting temporal dynamics in the immunological effects of moderate exercise [23]. A study examining 30 min of treadmill walking (6 km/h) in healthy but sedentary undergraduate students found no acute immunological changes following single exercise sessions [4]. However, when this protocol was extended to 6 days per week for 3 weeks, significant immuno-potentiating adaptations emerged, including decreased blood levels of interleukin (IL)-12p40, a receptor antagonist of the cellular immunostimulatory cytokine IL-12 [4,24].

Gender-specific responses to moderate exercise interventions reveal important considerations for functional food applications. Recent evidence demonstrates that females exhibit distinct inflammatory and antioxidant responses during the menstrual cycle phases, with the follicular phase showing enhanced anti-inflammatory effects from polyphenol supplementation compared to the luteal phase [5]. Recent evidence shows that females exhibit distinct inflammatory and antioxidant responses during moderate exercise. A randomized controlled trial in healthy female endurance athletes reported that nitrate-rich beetroot juice supplementation significantly enhanced aerobic capacity and improved oxidative stress markers, supporting the concept that sex-specific physiological characteristics may create unique windows of responsiveness to nutritional interventions [6]. Furthermore, sex differences in substrate utilization during moderate exercise indicate that females may benefit more from lipid-based functional foods, while males show greater responsiveness to carbohydrate-amino acid combinations [7]. These findings emphasize the need for sex-stratified approach in evaluating functional food efficacy during moderate exercise protocols. 

The elderly population presents unique considerations for exercise and nutritional interventions due to age-related increases in chronic inflammation and oxidative stress [2]. A 12-week walking intervention (100 min/week) in previously sedentary elderly subjects demonstrated remarkable improvements in antioxidant status, including increased blood glutathione peroxidase activity, elevated thioredoxin levels, and enhanced total antioxidant capacity, accompanied by reduced oxidative stress markers and neutrophil-related inflammation [8]. Notably, these benefits occurred despite exercise volume below current guidelines (150 min/week), suggesting heightened sensitivity to exercise-induced adaptations in inactive elderly individuals [8].

**Figure 1:**
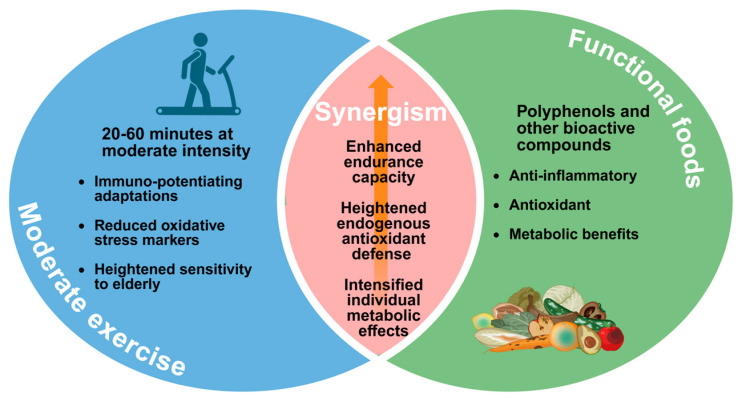
Synergistic benefits of combining moderate exercise with functional foods. Moderate exercise and functional foods each confer immunological, antioxidant, and metabolic advantages; when combined, they synergistically enhance endurance capacity, elevate endogenous antioxidant defenses, and intensify beneficial metabolic adaptations. Created in BioRender. Ma, S. (2025) https://BioRender.com/hlzqo3a (accessed on 20 November 2025).

Animal models provide valuable mechanistic insights into the synergistic effects of moderate exercise and functional foods. An 8-week study combining voluntary wheel running with black ginger (Kaempferia parviflora) administration in mice revealed enhanced endurance capacity and increased spontaneous running distance [9]. Molecular analysis showed activation of the Nrf2 (Nuclear factor erythroid 2-related factor 2)/ARE (Antioxidant Response Element) pathway in skeletal muscle, with upregulated gene expression of superoxide dismutase (*SOD*) 1 and 3, and components of the thioredoxin system. These changes resulted in increased antioxidant capacity in both blood and skeletal muscle [9]. Another study showed that combining exercise with grape polyphenol supplementation produced a synergistic effect, enhancing endurance by promoting muscle lipid oxidation and reducing glycogen utilization [10]. Such findings demonstrate how functional foods can amplify the beneficial effects of moderate exercise through targeted molecular mechanisms. Figure 1 summarizes the synergistic effects of moderate exercise combined with functional food intake.

Emerging biomarkers provide unprecedented sensitivity for detecting subtle effects of functional foods during moderate exercise. Circulating cell-free DNA (cfDNA), released from cells undergoing stress or death, serves as a real-time indicator of cellular turnover even during low-intensity exercise. Recent studies demonstrate that 30 min of walking at 50–60% VO_2_ max increases cfDNA by 1.5-2-fold [11]. This suggests protective effects at the cellular level not detectable through traditional markers.

Extracellular vehicles (EVs) and exosomes represent novel communication pathways between tissues during exercise. Moderate exercise induces a distinct exosome signature characterized by increased miR-21 and miR-146a content, which mediate anti-inflammatory signaling [12]. Functional foods, particularly omega-3 fatty acids and curcumin, have been shown to modify exosome cargo, enhancing their anti-inflammatory potential. A proteomics analysis revealed that 8 weeks of moderate exercise combined with quercetin supplementation altered exosomal proteins involved in muscle adaptation and mitochondrial biogenesis [13].

Continuous glucose monitoring (CGM) technology now enables real-time metabolic assessment during moderate exercise interventions. Recent data indicate that even non-diabetic individuals show glycemic variability during exercise that correlates with fatigue perception and recovery quality [14]. Functional foods that stabilize glucose excursions, such as resistant starch or chromium picolinate, demonstrate improved exercise enjoyment and adherence rates in sedentary populations initiating moderate exercise programs [15].

## 3. Intense Exercise

Table 1 summarizes key advantages, limitations and typical biomarkers associated with common exercise models used to assess food functionality in humans and rodents.

Intense exercise, exemplified by marathons, triathlons, and exhaustive training sessions, creates substantial physiological stress characterized by muscle damage, organ dysfunction, inflammation, and oxidative stress [16,17,18,19]. This exercise domain has traditionally been the focus of antioxidant supplementation strategies, though recent research has revealed unexpected complexities in these interventions [20,21].

The “antioxidant paradox” represents a critical consideration in sports nutrition. High-dose antioxidant supplementation, including vitamin C, has been shown to potentially impair training adaptations and, in some cases, exacerbate exercise-induced muscle damage [1,22,25,26]. Moreover, supplementation with vitamins C and E has been reported to attenuate key skeletal muscle adaptations to training, such as SOD and mitochondrial transcription factor A [22]. This paradoxical effect highlights the importance of reactive oxygen species (ROS) as signaling molecules essential for exercise-induced adaptations, challenging simplistic approaches to antioxidant supplementation [25,27].

### 3.1. Polyphenols and Other Bioactive Compounds

Polyphenols offer multi-targeted effects beyond simple antioxidant activity, including anti-inflammatory and metabolic benefits. Research with taheebo polyphenol demonstrated improved endurance through enhanced muscle glycogen metabolism in exhaustive exercise mouse models [28,29]. Furthermore, the beneficial effects of polyphenols have also been supported by clinical studies, reinforcing their potential as promising nutritional interventions for exercise performance and recovery [30,31,32,33,34]. However, the effects of polyphenols can be highly compound-specific and dose-dependent [25]. A cautionary example comes from studies with genistein, an isoflavonoid abundant in soybeans [35]. Despite its high antioxidant capacity [26,27,36], genistein administration in mice subjected to exhaustive exercise resulted in unexpected hepatotoxicity, with increased liver oxidative stress markers and suppressed skeletal muscle antioxidant gene expression, ultimately impairing exercise performance [36]. Similarly, (−)-Epigallocatechin-3-gallate (EGCG), the principal bioactive compound in green tea, demonstrates dual behavior as either an antioxidant or a prooxidant depending on the dose and the biological environment. At high doses, EGCG has also been reported to induce hepatotoxicity [37].

These contrasting findings emphasize the importance of careful compound selection and dose optimization. The development of highly bioavailable formulations has helped address some of these challenges. Nano-processed curcumin, achieving 185-fold increased absorption compared to standard formulations, has shown promise in preventing exercise-induced oxidative stress without interfering with adaptations [38]. Studies demonstrated that this enhanced curcumin formulation prevented oxidative stress during submaximal exercise (1 h run at 65% of maximal oxygen uptake (VO_2_ max) without causing muscle damage [39], and eccentric-exercise-induced muscle damage as well [40]. When mice were administered after downhill running, it reduced next-day oxidative stress while suppressing inflammatory cell infiltration and ROS production in the skeletal muscle [41].

**Figure 2:**
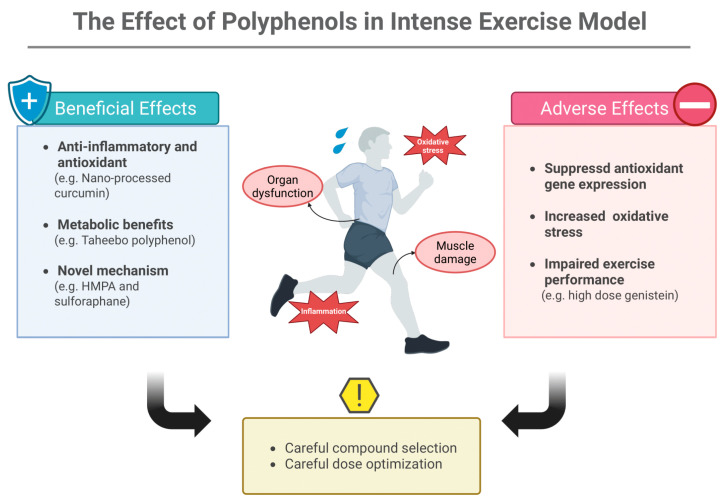
The Effect of Polyphenols in Intense Exercise Model. Polyphenols exert both protective and harmful actions during intense exercise: while certain compounds support anti-inflammatory, antioxidant, and metabolic adaptations, others may suppress antioxidant gene expression, increase oxidative stress, or impair performance at high doses. Appropriate compound selection and dose optimization are essential to maximize benefits and avoid adverse effects. HMPA: 3-(4-hydroxy-3-methoxyphenyl)propionic acid. Created in BioRender. Ma, S. (2025) https://BioRender.com/vx3cxfq (accessed on 20 November 2025).

Emerging research has identified novel mechanisms through which functional foods support intense exercise performance. The intestinal metabolite 3-(4-hydroxy-3-methoxyphenyl)propionic acid (HMPA), derived from polyphenol metabolism, not only increased antioxidant capacity and reduced oxidative stress but also enhanced muscle strength by promoting fast-twitch fiber formation via the insulin-like growth factor-1 (IGF-1) pathway [42]. Similarly, sulforaphane from broccoli sprouts has demonstrated protective effects against exhaustive exercise-induced inflammation and organ damage through Nrf2-mediated induction of antioxidant enzymes [43,44]. Another study reported that polyphenol extracts from Lonicera caerulea berries alleviate exercise-induced fatigue and improve performance in mice by reducing oxidative stress via the PKCα-Nox2/Nox4 pathway and enhancing mitochondrial biogenesis through the AMPK-PGC1α-NRF1-TFAM axis [45]. Figure 2 summarizes the evidence of polyphenols’ function in intense exercise regimens.

**Figure 3:**
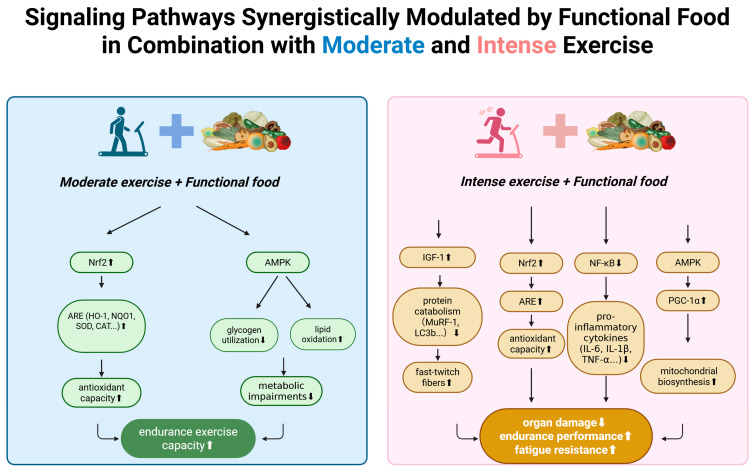
Synergistic modulation of signaling pathways by functional food and exercise. Functional foods enhance key molecular responses to moderate and intense exercise, including Nrf2-ARE antioxidant signaling, AMPK-mediated metabolic regulation, IGF-1–related muscle remodeling, and NF-κB suppression, collectively supporting improved endurance and reduced exercise-induced stress. Nrf2: Nuclear factor erythroid 2–related factor 2. ARE: Antioxidant Response Element. HO-1: Heme oxygenase-1. NQO1: NAD(P)H quinone dehydrogenase 1. SOD: Superoxide dismutase. CAT: Catalase. AMPK: AMP-activated protein kinase. IGF-1: Insulin-like growth factor-1. MuRF-1: Muscle RING-finger protein-1. LC3b: Microtubule-associated protein 1 light chain 3 beta. NF-κB: Nuclear factor kappa B. IL-6: Interleukin-6. IL-1β: Interleukin-1 beta. TNF-α: Tumor necrosis factor-alpha. PGC-1α: Peroxisome proliferator-activated receptor gamma coactivator-1 alpha. ↑: up regulation. ↓: down regulation. Created in BioRender. Ma, S. (2025) https://BioRender.com/7i6b8pw (accessed on 20 November 2025).

Metabolic approaches offer additional strategies for managing intense exercise stress. Ketogenic diets and medium-chain fatty acids have shown anti-inflammatory and antioxidant effects while improving endurance in animal models [46]. These interventions appear to work by improving energy metabolism efficiency, thereby reducing metabolic stress and associated ROS production. Both exercise and dietary compounds activate or inhibit conserved cellular signaling networks. Table 2 provides examples of compounds, their molecular targets and observed effects. Figure 3 integrates recent insights into key pathways (Nrf2–Keap1, NF-κB, AMPK, IGF-1/mTOR, SIRT1/PGC 1α) and their modulation by exercise and nutrition. 

### 3.2. Timing Strategies for Intense Exercise

The temporal dynamics of nutrient availability critically influence functional food efficacy during intense exercise. Recent pharmacokinetic studies using isotope labeling reveal distinct absorption windows that optimize performance benefits. Pre-exercise loading (2–4 h) allows for complete gastric emptying and peak plasma concentrations coinciding with exercise onset [51]. Nitrate supplementation shows optimal effects at 2–3 h pre-exercise, achieving peak plasma nitrite levels [52]. Polyphenols require longer lead times at muscle, with quercetin showing maximal at 1–3 h post-ingestion in circulation [53].

During-exercise supplementation feasibility depends on exercise intensity and gastrointestinal tolerance. Above 75% VO_2_ max, splanchnic blood flow reduces by 60–80%, severely limiting absorption [54]. However, novel delivery systems including hydrogels and nanoencapsulation maintain nutrient release at lower intensities. Branched-chain amino acids (leucine, isoleucine, valine) consumed during exercise below lactate threshold reduces central fatigue without gastrointestinal distress [55].

The post-exercise window represents a critical period for recovery optimization. The traditional “anabolic window” concept has evolved to recognize multiple phases: immediate (0–30 min) for glycogen replenishment, early (30 min–4 h) for protein synthesis, and extended (4–24 h) for inflammation resolution [56].

### 3.3. Environmental Stressors and Intense Exercise Models

The combination of environmental stressors with intense exercise creates unique models for evaluating functional food efficacy under extreme conditions. Heat stress (35–40 °C, 50–60% humidity) combined with exhaustive exercise amplifies oxidative stress by 3–4-fold compared to thermoneutral conditions [57]. Recent investigations demonstrate that pre-loading with specific polyphenols, particularly methylated catechins from green tea, enhances heat shock protein expression and improves thermal tolerance. Athletes supplemented with 600-mg EGCG daily for 7 days before heat-stress exercise showed 15% lower core temperature rise and 25% improved time to exhaustion [58].

Cold exposure exercise models (5–10 °C) reveal distinct nutritional requirements, with brown adipose tissue activation increasing metabolic demands [59]. Capsaicin and other thermogenic compounds demonstrate synergistic effects with cold-induced metabolism, enhancing fat oxidation during submaximal exercise [60]. Interestingly, antioxidant requirements paradoxically decrease in cold conditions due to reduced mitochondrial ROS production, suggesting environment-specific supplementation strategies [61].

Simulated altitude (2500–4000 m equivalent) combined with intense exercise provides insights into hypoxic stress management. Recent studies using normobaric hypoxic chambers demonstrate that nitrate supplementation (6–8 mmol/day) partially compensates for reduced oxygen availability, improving VO_2_ max at altitude [62]. Iron-vitamin C co-supplementation shows enhanced efficacy at altitude, with greater improvements in hemoglobin mass compared to sea level training [63,64,65]. These models are particularly relevant for athletes preparing for high-altitude competitions or training camps.

## 4. Training Models: Optimizing Adaptation and Recovery

Training represents a unique challenge in sports nutrition, as interventions must support both acute recovery and long-term adaptations. Different training modalities create distinct nutritional demands, requiring targeted approaches for optimal outcomes.

### 4.1. Resistance Training and Muscle Adaptation

Leucine and its metabolites play crucial roles in muscle protein synthesis and hypertrophy. β-Hydroxy-β-methylbutyrate (HMB), a leucine derivative, has evolved from a compound with limited efficacy due to poor absorption to a promising intervention through the development of HMB free acid (HMB-FA) [66,67,68]. A 6-week double-blind, randomized controlled trial demonstrated that 3-g HMB-FA daily, consumed immediately after resistance training, significantly increased muscle strength compared to placebo. This timing-specific effect appears related to exercise-induced secretion of growth hormone and IGF-1, creating an optimal anabolic environment [69].

**Age-Specific Training Adaptations:** Youth athletes (<18 years) present unique considerations due to ongoing growth and development. Peak height velocity, occurring around age 12 in females and 14 in males, creates windows of heightened nutritional demands [70]. During this period, protein requirements increase to 1.2–2.0 g/kg/day of protein, depending on the type of sport and training intensity [71]. Calcium and vitamin D become critical, with supplementation (1200 mg Ca + 1000 IU D3) reducing stress fracture risk in adolescent runners [72]. Dietary creatine intake among U.S. children and adolescents (2–19 years) averages 1.07 ± 1.07 g/day, with higher intake (≥1.5 g/day) associated with greater height, weight, and BMI, and each additional 0.1 g/day predicting ~0.3–0.6 cm taller stature, suggesting a potential role in growth regulation [73].

Sarcopenia is driven in part by age-related anabolic resistance—characterized by blunted muscle protein synthesis due to impaired signaling, reduced amino acid delivery, and increased splanchnic retention—exacerbated by obesity and inactivity, with evidence suggesting that older adults require higher protein/leucine doses to overcome this deficit [74]. Endurance exercise activates PGC-1α–mediated mitochondrial biogenesis, and emerging evidence suggests that PQQ (≈20 mg/day), through redox and energy metabolism pathways, may synergize with exercise to enhance mitochondrial density and function, though human data remain scarce [75]. Long-term collagen peptide supplementation (10–20 g/day, 6–9 months) improves activities of daily living, pain, and mental health scores in middle-aged active adults, with additional physical health benefits observed in females [76].

Plyometric training, characterized by explosive movements that enhance power and athletic performance [77], induces substantial muscle damage and oxidative stress. HMB-FA supplementation has proven effective in mitigating these negative effects while maintaining performance benefits [78]. Additionally, capsaicin (12 mg) has emerged as an interesting intervention for plyometric training, preventing delayed-onset muscle soreness while enhancing jump performance through transient receptor potential vanilloid 1(TRPV1) channel-mediated anti-inflammatory effects and enhanced energy production [79].

**Gut–Muscle–Brain Axis in Training**: The bidirectional communication between gut, muscle, and brain profoundly influences training adaptations and performance [80]. Gut-derived metabolites, particularly short-chain fatty acids (SCFAs), cross the blood–brain barrier and modulate central fatigue mechanisms [81]. Athletes exhibit greater gut microbial diversity and higher fecal SCFAs (butyrate, propionate), with taxa such as *Akkermansia* and *Veillonella* enriched, supporting enhanced energy metabolism, recovery, and immune regulation compared to sedentary individuals [82].

Mental fatigue, induced by prolonged cognitive tasks, reduces subsequent physical performance by 15–25% through altered perception of effort. The gut–brain–muscle axis links microbiota, neurotransmitters, and metabolism, with optimal gut health enhancing nutrient absorption, energy availability, immune function, and mental resilience to support peak athletic performance [80]. In elite wrestlers, combining L-theanine (≈3 mg/kg) with caffeine (≈3 mg/kg) enhanced strength, endurance, and Stroop test performance while lowering anxiety and side effects compared to caffeine alone, suggesting this blend more reliably preserves both cognitive and physical performance [83]. Recent fMRI studies reveal this combination maintains prefrontal cortex activation during combined mental-physical challenges [84]. A study in collegiate female swimmers found that 6-week supplementation with *Bifidobacterium longum* 35,624 (1 billion CFU/day) improved sport recovery scores and cognitive outlook without altering performance or immune markers [85]. The gut–brain axis also influences exercise-induced neuroplasticity, with certain probiotics enhancing BDNF production and potentially accelerating motor learning [86].

### 4.2. Endurance Training and Gut–Immune Interactions

Intense endurance training can compromise intestinal barrier function, leading to increased permeability “leaky gut”, systemic inflammation, and performance decrements [1]. Novel interventions targeting this gut–immune axis have shown promising results. Hyperimmune milk-derived immune proteins, obtained from cows vaccinated against specific pathogens, demonstrated protective effects in young runners [87]. Following 8 weeks of supplementation, runners maintained renal urinary concentrating ability after 3000 m time trials, indicating better hydration status. More importantly, exercise-induced elevations in intestinal injury markers (I-FABP), IL-1β, and TNF-α were significantly attenuated, suggesting preservation of intestinal barrier function [87]. Emerging evidence suggests that these effects may be mediated through beneficial modulation of the gut microbiota.

Direct manipulation of the gut microbiome through probiotic and prebiotic combinations has yielded sport-specific benefits. Sport-specific interventions have shown efficacy in other disciplines: carob polyphenols in taekwondo athletes increased antioxidant enzyme activities (SOD and catalase), reduced lipid peroxidation, and improved kick frequency after 6 weeks of supplementation [88]. However, not all interventions translate to performance benefits—beetroot juice supplementation in volleyball players prevented muscle soreness but failed to enhance jumping ability [89], highlighting the importance of selecting appropriate outcome measures.

## 5. Future Perspectives: Toward Personalized and Synergistic Approaches

The field of exercise nutrition is evolving toward more sophisticated, personalized approaches. Several key trends are shaping future directions:

### 5.1. Technological Advances in Bioavailability

Beyond traditional biomarkers, multi-omics integration provides systems-level understanding of exercise–nutrition interactions. Metabolomics profiling identifies over 500 exercise-responsive metabolites, with functional foods modulating specific pathways [90]. Real-time mass spectrometry, now portable for field testing, enables immediate metabolic phenotyping. Recent developments in sweat analysis using wearable sensors continuously monitor electrolytes, lactate, glucose, and cortisol, allowing dynamic supplementation adjustments [91]. Artificial intelligence algorithms analyzing these multi-dimensional datasets predict individual responses with 83% accuracy, enabling truly personalized nutrition prescriptions [92]. Digital twins—computational models of individual physiology—simulate supplement responses before implementation, reducing trial-and-error approaches [93].

### 5.2. Microbiome-Targeted Interventions

Recognition of the gut microbiota’s role in exercise adaptation, immune function, and metabolism is driving development of next-generation probiotics, prebiotics, and symbiotic specifically designed for athletic populations. Recent metagenomic studies reveal that elite athletes harbor distinct microbial signatures, with increased abundance of *Veillonella* atypical, which metabolizes lactate to propionate, potentially enhancing endurance [94]. This discovery has led to development of performance-specific probiotic formulations targeting metabolic pathways unique to exercise.

Strain-specific effects are now well-documented, moving beyond generic probiotic recommendations. *Lactobacillus plantarum* PS128 specifically increases dopamine and serotonin production, improving exercise motivation and reducing perceived exertion [95]. *Bifidobacterium breve* BR03 combined with *Streptococcus thermophilus* FP4 reduces exercise-induced IL-6 elevation by 43% [96].

Prebiotic strategies have evolved beyond simple fiber supplementation to targeted microbiome modulation. Microbial fermentation metabolites such as SCFAs exert wide-ranging effects on gut and systemic physiology, whereas protein-derived metabolites occur at non-toxic levels; however, current evidence is insufficient to use fecal metabolite concentrations as reliable markers of prebiotic efficacy, highlighting the need for integrated metabolomics–metagenomics approaches and flux-based analyses [97]. Polyphenol-rich prebiotics, particularly pomegranate ellagitannins and green tea catechins, create a selective advantage for *Akkermansia muciniphila*, associated with improved metabolic efficiency and better running economy [98].

Postbiotic applications represent the newest frontier, offering standardized bioactivity without viability concerns. Heat-killed *Lactobacillus paracasei* MCC1849 maintains anti-inflammatory effects while eliminating refrigeration requirements, crucial for traveling athletes [99]. Extracellular vesicles from certain probiotics can deliver bioactive compounds to intestinal cells and enhance gut barrier integrity more effectively than whole probiotic cells. For example, vesicles from *Limosilactobacillus reuteri* reduced pathogen-induced gut leakage by up to 65% in vitro, demonstrating a potent barrier-protective effect [100]. Taking SCFAs directly (such as tributyrin at 3–6 g/day) can bypass the need for microbial fermentation and provide performance benefits. SCFAs are readily absorbed and used in muscle; studies show they enhance lipid oxidation and spare glycogen during exercise [101].

Filtered, cell-free supernatants containing probiotic metabolites (postbiotics like bacteriocins) can confer immune benefits without needing live colonization. In athletes, probiotic supplementation is linked to reduced upper respiratory tract infection incidence by ~27–50% [102]. For example, over a winter training period only 35% of athletes on probiotics reported any cold symptoms vs. 79% on placebo [102]. This immediate immune protection suggests that even non-living probiotic products could similarly lower infection risk (around one-third reduction) in high-stress periods for athletes.

Exercise training itself modulates gut microbial composition. Regular vigorous exercise (e.g., 6 weeks of interval training) is associated with increased gut microbe diversity [103]. Athletes show a significantly wider range of gut microbiota than non-athletes. One study found elite athletes had much higher microbial diversity than BMI-matched controls [104]. Prolonged extreme exercise can temporarily *reduce* gut diversity and beneficial bacteria. For instance, after a 217 km ultramarathon, an obese runner’s gut α-diversity dropped and symbiotic microbes decreased, while harmful bacteria spiked [105]. This acute dysbiosis post-ultra supports reports of 20–30% diversity reductions transiently after ultra-endurance events. Targeted restoration (through diet or probiotics) is often needed to rebound the microbiome to pre-race status [105]. Emerging research uses machine learning to analyze athletes’ microbiomes for performance insights. Early studies in animal models show gut profiles can predict endurance capacity. In humans, high-throughput “-omics” data are being used to predict traits like substrate utilization or fatigue [106]. In short, the exercise–microbiome relationship is bidirectional: training alters the microbiome, and analyzing the microbiome can potentially guide individualized training and nutrition in the near future.

### 5.3. Combination Strategies—Interactions of Functional Foods and Drugs

Large doses of Vitamins C (>1 g) and E (>400 IU) can *impair training gains* by blunting exercise-induced oxidative signaling. Research shows antioxidant-supplemented athletes had significantly smaller increases in mitochondrial proteins (e.g., COX4 up +59% in placebo vs. −13% with vitamins) and PGC-1α (no rise with vitamins) after training [107]. In short, excessive antioxidants reduced markers of mitochondrial biogenesis by ~25–30%, corroborating the caution that high-dose C/E dampen endurance training benefits [108].

Iron supplements can inhibit zinc absorption when co-administered, due to competitive uptake. Clinical studies show high iron intakes negatively affect zinc absorption in adults [109]. To avoid this, athletes are advised to separate iron and zinc dosing to prevent one micronutrient impairing the other’s uptake.

Calcium (from dairy or supplements) interferes with non-heme iron absorption [110]. Even a single glass of milk or calcium tablet can significantly reduce iron uptake [111]. Caffeine alone can boost performance but mixing it with ephedrine (a stimulant once in some weight-loss supplements) amplifies cardiovascular strain. This combo has been associated with elevated heart rate, blood pressure, and even arrhythmia or stroke in case reports [112]. Due to such risks, ephedrine-containing supplements are banned in sports. Athletes are warned that caffeine’s effects on the heart and blood pressure are markedly potentiated by ephedrine.

### 5.4. Precision Nutrition

Gilbert’s syndrome, present in ~5–10% of Caucasians, is a mild genetic bilirubin metabolism disorder (low UGT1A1 enzyme) that slows glucuronidation in the liver. Athletes with Gilbert’s have reduced capacity to conjugate compounds—meaning they may clear some supplements more slowly [113]. They tend to be more sensitive to substances requiring glucuronidation (e.g., curcumin, quercetin). For instance, large doses of curcumin or other polyphenols might lead to higher plasma levels in Gilbert’s patients because the usual glucuronidation pathway is only ~30% as active [114]. Such individuals may benefit from lower doses or spacing out doses of glucuronidated supplements, and focusing on liver-supportive foods (cruciferous veggies, fermented foods, etc.).

A common polymorphism in the MTHFR gene (C677T) reduces the conversion of folic acid to active folate [115]. About 40% of people are heterozygous for an MTHFR variant that can lead to elevated homocysteine and lower B-vitamin levels [115]. Those with MTHFR mutations often do better with methylated B-vitamins (e.g., L-5-methylfolate, methylcobalamin) instead of standard folic acid or B12. Indeed, studies show supplementing with 5-MTHF (methyl-folate) improves folate status more effectively than folic acid in MTHFR mutation carriers. Athletes with known MTHFR issues use activated forms of B9/B12, ensure adequate B6 and riboflavin (which support the pathway), and monitor homocysteine—this can optimize their energy and recovery, given B-vitamin roles in methylation and endurance performance.

Ultimately, individual responses vary based on genetics, health status, and environment. Personal tolerance must be established gradually. Sport nutritionists emphasize slow dose escalation and close monitoring when introducing any potent functional food or supplement. For example, with caffeine or beta-alanine or bicarbonate, some athletes respond strongly or get GI upset, while others do not [116]. Starting with a low dose in training and tracking performance and side effects is crucial to find one’s safe and effective range. Additionally, new wearable biosensors (glucose monitors, etc.) can help tailor nutrition in real-time (e.g., adjusting fuel intake to one’s blood sugar trends) [116,117]. The overarching principle is personalization: what works for one athlete might need modification for another. Gradual self-experimentation under professional guidance ensures safety while optimizing efficacy.

## 6. Conclusions

The selection of appropriate exercise models is crucial for evaluating food functionality. Moderate exercise reveals long-term anti-inflammatory and antioxidant benefits of functional foods, though effects may require weeks to manifest. Intense exercise models expose the complexity of nutritional interventions, exemplified by the antioxidant paradox, while highlighting successful strategies like nano-processed compounds and metabolic modulators. Training models demonstrate the importance of matching interventions to specific athletic demands, with emerging evidence supporting gut–immune axis modulation as a promising approach.

Future translational research should prioritize three key areas: (1) development of point-of-care biomarker testing enabling real-time personalization of exercise–nutrition interventions based on individual responses; (2) clinical validation of microbiome-targeted approaches, particularly sport-specific probiotic formulations and their integration into training periodization; (3) implementation of digital health technologies combining wearable sensors with AI algorithms to optimize supplement timing and dosing.

For clinical applications, immediate opportunities exist in sports medicine clinics for nano-formulated supplements with enhanced bioavailability, in geriatric care for combined exercise–polyphenol interventions targeting sarcopenia, and in post-COVID-19 rehabilitation programs integrating graduated exercise with targeted nutritional support. The convergence of precision medicine with exercise science promises to transform functional foods from general supplements into precisely prescribed interventions, ultimately achieving optimal balance between performance enhancement and health protection through evidence-based, personalized nutritional strategies.

## Figures and Tables

**Table 1:** Key advantages, limitations and typical biomarkers associated with common exercise models used to assess food functionality in humans and rodents.

Model (Species)	Advantages	Limitations	Typical Endpoints/Biomarkers
Treadmill running (rodent)	Controlled speed, incline and duration; reproducible workload; suitable for VO_2_ max and metabolic rate measurement	Forced exercise induces stress; unnatural gait; requires training and motivators (e.g., shocks)	VO_2_ max, lactate, cortisol, cytokines, antioxidant enzymes
Voluntary wheel running (rodent)	Low stress; reflects habitual physical activity; useful for long-term interventions	Low control over intensity; high variability; nocturnal running patterns	Distance run, activity patterns, basal cytokines, body weight
Endurance training (human)	Widely used protocols; enhances aerobic fitness and mitochondrial biogenesis; sensitive to antioxidant interventions	Requires long duration; may not address muscle hypertrophy; adaptation reduces inflammatory responses over time	VO_2_ max, lactate threshold, antioxidant enzymes, cytokines
Resistance training (human)	Increases muscle mass and strength; stimulates mTOR–IGF-1 pathway; relevant for sarcopenia	Skill-dependent; difficult to standardize across participants; limited improvements in cardiovascular fitness	1-RM, muscle cross-sectional area, IGF-1, mTOR phosphorylation, creatine kinase
High-intensity interval/eccentric exercise (human)	Elicits pronounced oxidative stress and inflammation; useful for testing anti-inflammatory compounds	May cause muscle damage and soreness; not sustainable for certain populations	IL-6, TNF-α, creatine kinase, DOMS, oxidized glutathione

**Table 2:** Examples of functional compounds, their molecular targets and observed effects.

Functional Compound	Major Molecular Target(s)	Evidence and Effects
Resveratrol	Activate Nrf2–Keap1 pathway; inhibit NF-κB; promote mitochondrial biogenesis via SIRT1/PGC-1α	Resveratrol enhances mitochondrial function and insulin sensitivity, is associated with a beneficial effect on the sports performance [47].
Curcumin	Suppresses NF-κB and COX-2; activates Nrf2/HO-1; reduces NLRP3 inflammasome	Curcumin supplementation attenuates exercise-induced increases in IL-6 and lipid peroxidation; combining curcumin with endurance training reduced muscle soreness and oxidative stress [38,39].
Omega-3 PUFAs	Incorporate into membranes; reduce proinflammatory eicosanoids	n-3 PUFAs mitigate delayed-onset muscle soreness and reduce free radical production [48].
Dietary nitrate (beetroot juice)	Increases nitric oxide via nitrate–nitrite–NO pathway; improves mitochondrial efficiency; lowers VO_2_ cost	At altitude, nitrate supplementation improved 16.1 km cycling performance, but results are inconsistent [49].
HMB	β-hydroxy-β-methylbutyrate (HMB) activates mTOR and suppresses proteolysis	HMB reduces markers of muscle damage and enhances recovery after intense training [50].

## Data Availability

No new data were created or analyzed in this study. Data sharing is not applicable to this article.
